# The Plight of a Bombay Hospital

**Published:** 1921-09-24

**Authors:** 


### The Plight of a Bombay Hospital.

In a recent number of the *Times of India* appeared a remarkable letter, signed J. R. Roberts, President of the Western India Automobile Association, drawing attention to the condition of the native hospital of Bombay. "We have" (he says) "a serious and crying necessity in improving the accommodation and treatment of the hundreds of citizens of Bombay who are mangled by numerous motor-car, machinery, and accidents in the streets, the docks, and factories of this city. A visit to the wards of the Gokuldas Tejpal (Native General) Hospital is a revelation. Nothing can be more heartrending than the mangled humanity that fills its beds, and the toll of those brought in dead or dying. This institution is asked to accommodate, to treat, and to cure an amount of human suffering that is almost beyond words to describe. Yet the equipment, according to modern ideas, is most inadequate, the hospital is starved for funds (that, however, is no fault of the Government, as it cannot give more) . . . Massage and electro-therapeutics have done so much for the maimed, yet there is no such department at the Gokuldas Hospital. X-rays are not employed, though an apparatus of now doubtful utility and sadly in want of repair does exist, and there is no expert to work this important aid to diagnosis and treatment. . . . It would be a suitable memorial of the Prince of Wales' visit for the Gokuldas Hospital to be refurnished, re-equipped, and kept up to date. A rough estimate would mean something like three lakhs of rupees, which this generous city could surely find for its mangled citizens."

According to the latest available statistics, the hospital possesses 120 beds, with the staff of one house surgeon and four native nurses.

